# The patient needs assessment in cancer care: identifying barriers and facilitators to implementation in the UK and Canada

**DOI:** 10.1007/s00520-020-05542-6

**Published:** 2020-06-04

**Authors:** Susan Williamson, Thomas F. Hack, Munirah Bangee, Valerio Benedetto, Kinta Beaver

**Affiliations:** 1 University of Central Lancashire, Preston, UK; 2 College of Nursing, Rady Faculty of Health Sciences, University of Manitoba, Winnipeg, Canada

**Keywords:** Cancer, Cancer survivorship, Recovery package, Holistic needs assessment, COMPASS

## Abstract

**Purpose:**

Personalised information and support can be provided to cancer survivors using a structured approach. Needs assessment tools such as the Holistic Needs Assessment (HNA) in the UK and the Comprehensive Problem and Symptom Screening (COMPASS) questionnaire in Canada are recommended for use in practice; however, they are not widely embedded into practice. The study aimed to determine the extent to which nurses working in cancer care in the UK and Manitoba value NA and identify any barriers and facilitators they experience.

**Method:**

Oncology nurses involved in the care of cancer patients in the UK (*n* = 110) and Manitoba (*n* = 221) were emailed a link to an online survey by lead cancer nurses in the participating institutions. A snowball technique was used to increase participation across the UK resulting in 306 oncology nurses completing the survey in the UK and 116 in Canada.

**Results:**

Participants expressed concerns that these assessments were becoming bureaucratic “tick-box exercises” which did not meet patients’ needs. Barriers to completion were time, staff shortages, lack of confidence, privacy, and resources. Facilitators were privacy for confidential discussions, training, confidence in knowledge and skills, and referral to resources.

**Conclusion:**

Many busy oncology nurses completed this survey demonstrating the importance they attach to HNAs and COMPASS. The challenges faced with implementing these assessments into everyday practice require training, time, support services, and an appropriate environment. It is vital that the HNA and COMPASS are conducted at optimum times for patients to fully utilise time and resources.

## Background

The United Kingdom (UK) and Canada have similar public healthcare systems which are struggling to manage the needs of increasing numbers of patients diagnosed and treated for cancer [1, 2]. In 2010, a report published by the National Cancer Survivorship Initiative (NCSI) in the UK stated that people who have been diagnosed and treated for cancer face many long-term difficulties and need support and information to live well with and beyond the cancer diagnosis [3]. The NCSI advocated that supportive care for cancer survivors should be given equal importance to acute care, with a prominent focus on self-management [3]. The Cancer Strategy for England Report (2015), in line with the NCSI report, suggests that patients should be supported to manage their cancer effectively as a chronic condition [1, 4, 5]. In Canada, emotional distress has been branded as the “6th vital sign”, granting it prominence in oncology alongside the traditional five vital signs, and requiring cancer programs to implement appropriate distress screening protocols [6].

To ensure cancer survivors’ optimum health and wellbeing, a structured approach to assessing people’s physical, practical, emotional, spiritual, and social needs has been recommended in the UK and Canada. In the UK, the Holistic Needs Assessment (HNA) is a pivotal component in the development of a Recovery Package for people with cancer. The Recovery Package, which is a national initiative in the UK, is intended to not only improve communication about care needs between secondary and primary care but aims to provide people with the information and support they need to enable them to return to work, self-manage, and live their lives well [4, 7], [http://www.ncsi.org.uk/what-we-are-doing/the-recovery-package/]. Elements of the Recovery Package are as follows: Holistic Needs Assessment, Treatment Summary, Cancer Care Review, and Health and Wellbeing Clinics. Key time points suggested for conducting the HNA are at diagnosis, after completion of treatment, at discharge from routine follow-up, at episodes of disease recurrence, and at transition from active to palliative care [http://www.ncsi.org.uk/what-we-are-doing/the-recovery-package/]. In Canada, a guideline developed by the Canadian Partnership against Cancer and the Canadian Association of Psychosocial Oncology recommends that all cancer patients be routinely screened for distress, contributing problems and associated concerns [8]. Similar to the UK, this screening should take place at the initial visit, at appropriate intervals, and whenever clinically indicated, using brief, validated tools. In Manitoba, a Canadian province, distress screening is expected to be performed at every patient visit using the Comprehensive Problem and Symptom Screening (COMPASS) questionnaire. This is comprised of modified versions of the Edmonton Symptom Assessment Scale [9] and the Canadian Problem Checklist [6] which asks patients to indicate items that were a source of worry or concern during the previous week from seven domains (Physical, Practical, Emotional, Spiritual, Informational, Social/Family, and Dignity) [10]. In Manitoba, clerical staff ensure that patients complete the COMPASS, while nurses are responsible for using the COMPASS scores to respond according to the national guideline [8].

In the UK, there is an expectation that clinical nurse specialists (CNSs) working in cancer care will perform the HNA, as their role is to coordinate care, provide technical and psychosocial information, and emotional support [http://www.ncsi.org.uk/what-we-are-doing/the-recovery-package/] [11]. The information collated in the HNA should, in conjunction with a risk stratification strategy, inform the patients individual care pathway and ensure information and support is provided that is tailored to patients’ individual needs [12]. However, it is acknowledged that completion of the HNA is not widely embedded into practice [13, 14]. In Canada, there is no designated oncology professional for distress screening efforts, but usually clerical or nursing staff ask the patients to complete the screening questionnaires. In Manitoba, oncology nurses are responsible for using the COMPASS scores to open up a conversation with the patient and respond to distress according to national guidelines [8]. The guidelines state that patients who score high on distress should have a comprehensive assessment of their distress to identify the sources and magnitude of distress, and associated risk factors. Using a stepped-care approach, all patients should be offered education and support, followed by a tailored psychosocial intervention based on the intensity (mild, moderate, severe) of their distress. The clinical success of initial distress screening efforts is critically dependent on a subsequent, more comprehensive assessment of the patient, based on the patient’s COMPASS score.

A number of barriers have been identified that may prevent CNSs from carrying out a HNA including CNS shortages, lack of time, concern about causing distress to patients, lack of confidence in ability to answer difficult questions, the organisational culture being disease rather than patient focussed, and lack of training and support [15, 16]. In Canada, the perceived value of distress screening is mixed. One survey found that 38% of oncologists regard distress screening as impractical, and 43% of nurses and radiographers report that distress screening is not useful [17]. A second study found that once distress screening efforts were underway, staff were enthusiastic about the process and appreciated the associated training [18]. Others reported that distress screening provides a means of demonstrating quality care to patients and coordinating care [19].

Assessment of patients’ individual needs should be at the core of any self-management approach to care and it may be that this information is being collected through other means and not labelled or identified as a HNA or COMPASS evaluation. Conversely, institutions may be implementing the HNA/COMPASS successfully but not formally reporting on their progress; this remains unexplored. In the UK, patient access to CNSs varies geographically and by tumour site, which is problematic if a HNA is a pivotal aspect of planning a care pathway [13]. Tailored care pathways were being developed and implemented, but not always based on a Needs Assessment (NA). CNSs also felt that it was challenging to undertake risk stratification without a completed NA, compounding efforts to provide patients with tailored self-management care pathways.

The aim of this study was to determine the extent to which nurses working in cancer care in the UK and Manitoba value NA and identify any barriers and facilitators they experience.

## Study objectives

To identify:

1. What constitutes a NA at different localities, including the assessment time points
2. Whether oncology nurses perceive the assessment as beneficial for patients and a key component of care planning
3. What barriers/facilitators inhibit/assist oncology nurses in carrying out the assessment
4. Recommendations for incorporating the NA into routine, clinical practice

## Methods

A questionnaire was developed for completion online using Survey Monkey to establish whether nurses working in cancer care use a NA, when they use it, and the perceived barriers and facilitators to using it. Informal discussions with oncology nurses in the UK and Manitoba identified similar concerns about the patient needs assessments including the timing, frequency, and value of the assessment.

Items for inclusion in the survey were derived from the literature and informal discussions with nurses working in oncology, lead cancer nurses and patient groups in the UK and Manitoba which have similar practices and models of care. Although reliability and validity were not formally tested, questions were refined and tested by lead cancer nurses until they were satisfied that wording and questions were clear and in the right format. Small changes were made to each question, and some were altered to reflect differences in recommended practice in the UK and Manitoba. The questionnaire was then completed by two oncology nurses who were not going to participate in the online survey.

The final version included 24 questions, 19 closed with an “other” option if the closed responses did not represent their practice, and 5 open-ended. Respondents were asked if patients had a NA, the timepoints in the care pathway at which it was completed, how long it took to complete, and how the information collected was used. Other questions asked about training, barriers, and facilitators to conducting a NA including ticking concerns from the list in the COMPASS/HNA document that they found difficult to discuss with patients. Lastly, they were asked to indicate the type of hospital or community practice they worked in, which cancer group they worked with and the country they worked in.

### Sample and recruitment

Lead cancer nurses in the Lancashire and South Cumbria regions (LSC) in the UK and Manitoba were asked to forward an email with a link to the online survey to a convenience sample of all oncology nurses who had responsibility for completing a NA.

In addition, a snowball sampling technique was used to recruit CNSs working in cancer care across the UK through their national societies. The survey link was advertised through the UK Oncology Nursing Society, British Psychosocial Oncology society, and Macmillan Cancer Support newsletters/websites. In Manitoba, the link to the survey was distributed to the managers of oncology nursing of several regional health authorities who sent the survey link to CancerCare Manitoba nursing staff. The inclusion criteria were all nurses working in cancer care who would have responsibility for completing a Needs Assessment for patients, regardless of duration of work experience. Oncology nurses who did not have responsibility for completing a needs assessment were excluded. When potential participants opened the link to the survey, they were presented with information about the study. After reading the information, they could then choose whether or not to continue to the survey. This information page also provided contact details for the research team, assurances that participation was voluntary, and that responses would only be seen by the research team at the university. Consent was provided through anonymous participation. Data were collected between May 2017 and November 2017. Ethical approval was gained from the University of Central Lancashire in England, the Health Research Ethics Board at the University of Manitoba, and the Regional Health Authority Research Ethics Committees in Manitoba: Winnipeg, Southern Health, Interlake/Eastern, and Northern Health.

### Analysis

The quantitative data from closed responses were exported from Microsoft Excel into SPSS for Windows (version 22) and analysed using descriptive statistics to establish frequencies. Responses from the UK were cross tabulated with responses from Canada. For brevity, the number (n) and percentage of participants who gave specific answers are reported. The qualitative data from the responses to open-ended questions were uploaded into NVivo and analysed using content analysis [20].

## Results

Initially, 110 CNSs were invited to participate across LSC and 80 (73%) responded. A snowball technique was used for further recruitment, and 306 responses were received representing England, Scotland, Wales, and Northern Ireland. In Manitoba, 221 oncology nurses were invited to participate and 116 (52.5%) responded. Not all questions were answered by all, and totals represent the number of valid responses to questions. Many respondents used the “other” section of closed responses to explain or add to their forced choice. Representative quotations from these responses are provided throughout the results section.

### Sample characteristics

In the UK, most respondents worked in secondary care (90%, *n* = 187) with the remaining 10% (*n* = 28) working in primary care or in integrated posts. In Manitoba, all respondents worked in either secondary or quaternary care (100%), *n* = 116. All respondents worked across a broad range of cancer specialities so we can be confident that practice and issues described were not limited to one speciality.

### Completing and utilising the HNA/COMPASS

**Fig. 1:**
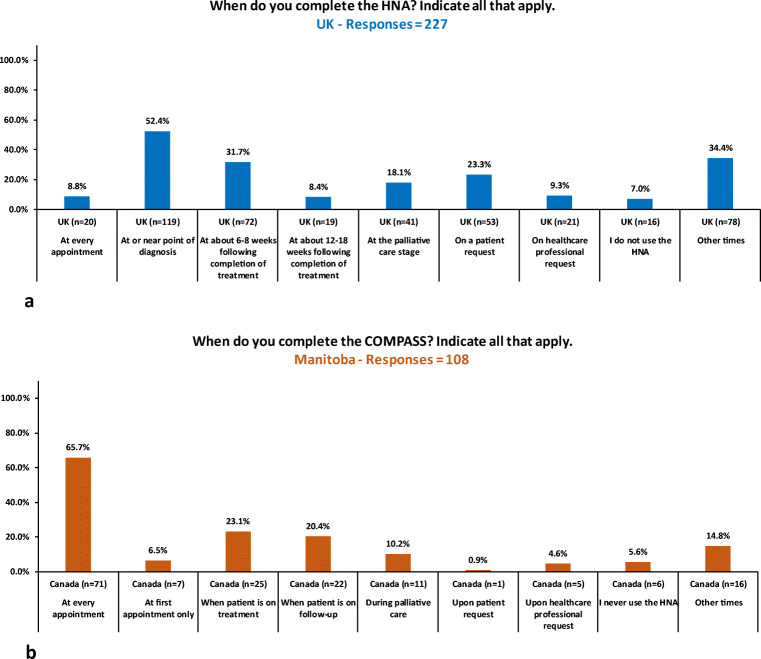
**a** Completion of the HNA. UK—Responses = 227. **b** Completion of the COMPASS. Manitoba—Responses = 108

Respondents reported that the majority of cancer patients had received a NA in the UK (96% *n* = 260), and in Manitoba (91%, *n* = 130). There were differences with the way the NA was completed; mainly face to face with patients in the UK (86%, *n* = 204) and self-completed by patients in Manitoba on arrival at the oncology clinic for discussion during their appointment (97%, *n* = 110). Slightly more than half of UK respondents (52%, *n* = 119, *N* = 227) completed the NA with patients at or near point of diagnosis, and only 9% (n = 20) completed the NA at every appointment (Fig. 1a). This contrasts with the much higher percentage of Manitoba respondents who discussed a NA at every appointment (66%, *n* = 71, *N* = 108) (Fig. 1b). In the UK, the rate of completion of the NA during follow-up decreased over time: 32% of respondents completed the NA at about 6–8 weeks following completion of treatment (*n* = 72), while only 8% of respondents completed the NA at about 12–18 weeks following completion of treatment (*n* = 19). During follow-up, the percentage of Manitoba respondents who completed COMPASS (20%, *n* = 22) did not differ greatly from the percentage of respondents who completed the HNA during treatment (23%, *n* = 25). In the palliative care stage, 18% of UK respondents completed a NA (*n* = 41), compared with just 10% of Canadian respondents (*n* = 11). The majority of respondents indicated that the NA was used to develop individualised care plans in the UK (89%, *n* = 213), while in Manitoba, it was used to inform psychosocial and family support service needs (77%, *n* = 86). The time points at which the HNA and COMPASS were completed is shown in Fig. 1a and b.

Information relating to the NA was collected through a combination of means, primarily through both electronic and paper records in the UK (39%, *n* = 91), and electronic records in Manitoba (75%, *n* = 85). Across the UK, 10% (n = 22) of respondents said it took them less than 10 min to go through the HNA checklist with 77% (*n* = 176) saying it took them more than 10 min. In Manitoba, 81% (n = 91) said the COMPASS took less than 10 min with 8% (*n* = 9) saying it took them more than 10 min.

### Training

Most respondents in the UK (145/244, 59%) and Manitoba (90/113, 80%) had received training on how to deliver the NA. The majority had found the training adequate (UK 89%, Manitoba 84%).

An open-ended question asked respondents to comment on what training would be useful. A total of 132 responses were received (*n* = 70 for the UK, *n* = 62 for Manitoba). The responses were collapsed into the following categories:

- General training on NA—such as observing someone experienced in NA, and training to develop care plans.
- Communication training—how to broach sensitive issues, and training to improve confidence in dealing with sensitive issues.
- How to implement the NA into practice, rather than a bureaucratic tick box exercise
- Following up on NA—Respondents were not sure what they could do with the information received from patients, or whom they could refer patients to if necessary.

All respondents were concerned that the NA would become a bureaucratic exercise, not allowing time to go through it with patients and provide the necessary support and information that patients need. The expectation of nurses to act upon the NA was an area respondents felt needed clarification. How to help patients once issues have been identified was a key area for many.

Why do we want to know and what are we offering to do to address the concerns patients raise? At the moment it seems like a tick box exercise, we ask people for information we have no plan or means to do much with (UK 48).

List of resources for the patient if they have identified a barrier to care or to their health (MB 102).

### Barriers and facilitators to completing needs assessments

**Fig. 2:**
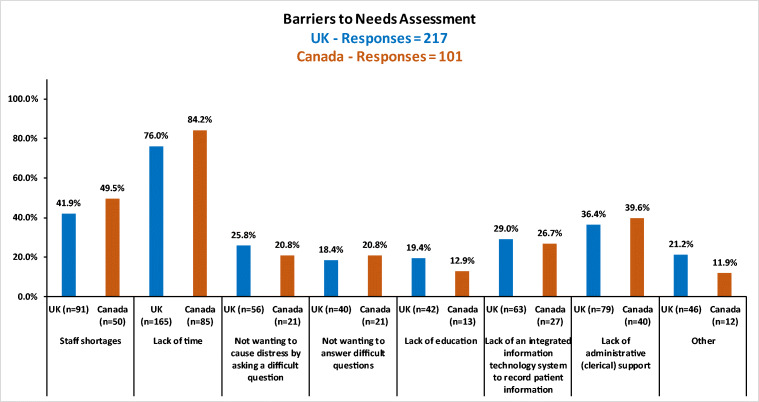
Barriers to needs assessment

Lack of time and staff shortages were the largest barriers to completing the NA in both groups of respondents (Fig. 2). In particular, the percentages of those who identified these problems as barriers were higher for the Manitoba respondents than for the UK respondents.

An open-ended question asking what additional COMPASS/HNA training respondents would find useful was answered by 72 (24%) respondents in the UK and 38 (23%) in Manitoba. Responses were collapsed into 4 main categories:

1. Lack of resources; including being unable to refer patients to appropriate support*.*
2. Timing of NA being inappropriate; respondents were not comfortable discussing some issues with patients at the time points suggested for completion of the NA.
3. Unsuitable environment; lack of privacy in clinics and wards and patients being unwilling to discuss sensitive issues in front of accompanying relatives were perceived as a barrier to personal conversations.
4. No process to map trends.
  I feel it is inefficient not being able to trend previous results to see if the level of pain/nausea etc. has been changing to help guide assessment and management (MB 64).
  I think information re: an overview of where to signpost patients to. Services change and although you identify a problem, I don’t always know where to go to with it (UK 300).

Respondents were asked to indicate on a checklist any issues from the COMPASS or HNA tool that they found difficult to discuss with patients. The majority found Sexuality, Financial issues, Emotional concerns, and Spirituality difficult to discuss with patients. Even though this question did not ask specifically about barriers, many respondents added that they found certain issues difficult to discuss because they did not know where to refer the patients if necessary. Only 5 respondents did not find any topics difficult to discuss with patients.

Facilitators to completing the HNA/COMPASS included suitable physical environments for private discussions (for matters of a sensitive nature) together with adequate training to enhance the knowledge of HNA/COMPASS and its implementation, and the availability of resources to refer patients for additional support.

Directory of services etc. that you can signpost pts to as appropriate when problems identified. Referral pathways to services (UK 143).

## Discussion

This study aimed to assess the extent to which oncology nurses perform a comprehensive, patient needs assessment of patient symptoms, problems, and needs, and identify barriers and facilitators to implementation of these assessments. We provide evidence that in the UK, the CNSs do complete the HNA but this is primarily carried out prior to and immediately after treatment, but not for all patients. Whereas in Manitoba, the COMPASS is completed by patients at every appointment but not always discussed with them by oncology nurses. In the UK, although other clinicians may conduct a HNA, CNSs are the clinicians who are recognised in policy documents in the UK as playing a key role in the provision of information and support to patients (http://www.ncsi.org.uk/what-we-are-doing/the-recovery-package/; http://www.macmillan.org.uk/Documents/AboutUs/Research/ImpactBriefs-ClinicalNurseSpecialists2014.pdf) [4, 11, 12]. It was clear that respondents in this study felt they had increasing and competing demands on their time. The Independent Cancer Taskforce reports that not only are numbers of CNSs insufficient in many cancer specialities but their time is frequently used inefficiently [4]. The respondents in this study cited lack of time to complete the HNA, and this may be why so many indicated that they did not routinely assess patients’ holistic needs at 12–18 months post treatment and prior to discharge. Indeed, this “lack of time” barrier prompted CancerCare Manitoba to ask clerical staff to ensure that patients complete the COMPASS routinely upon every clinic visit. Utilising clerical staff in this manner was intended to enable oncology nurses to spend time with patients, exploring their COMPASS responses and having conversations about identified problems and concerns. However, oncology nurses in Manitoba expressed equal concerns to their counterparts in the UK, citing lack of time to adequately address patient concerns and not knowing who to refer patients to if required.

Although most patients cope reasonably well post-treatment, a study of patients with head and neck cancer found significant numbers had unmet needs up to 5 years after completion of treatment [21]. These unmet needs covered a broad range of issues including tiredness and fatigue, worry and depression, physical concerns related to treatment, and fear of recurrence [21]. While some suggest that fear of recurrence diminishes over time, most agree that emotional concerns, worry, and depression continue for years post-treatment [21–24]. Moreover, the Cancer Patient Experience Survey reported that, while access to a CNS is the most important factor contributing to a positive patient experience, significant numbers do not receive the emotional support they need or the opportunity to discuss fears and worries [12]. The respondents in this study suggest that lack of clear referral pathways, lack of time, lack of privacy, and lack of confidence are the reasons patients have limited opportunities to discuss psychosocial issues. In Canada, this reality spawned a national training effort to instruct oncology nurses on how to respond therapeutically to patient reports of distress (anxiety, depression, fatigue, and pain). This comprehensive training program, including didactic online courses and reflective practice, significantly raised the confidence levels of nurses [25].

Other studies also report that lack of confidence, feeling over-burdened, and being ill prepared to deal with psychosocial distress are concerns for those tasked with completing the HNA [16, 24, 26, 27]. The respondents to this survey suggested that lack of privacy was a barrier for patients, either because relatives were present or because the clinic or ward environment was not conducive to personal discussions. Biddle et al. (2016) also suggested that some patients were unwilling, or found it difficult, to discuss psychosocial issues which will impact on the successful implementation of the recovery package in the UK and compliance with national guidelines in Canada, http://www.ncsi.org.uk/what-we-are-doing/the-recovery-package/ [8, 26].

### Implications for practice

Clinical staff need training, support, and clear referral pathways in order to feel confident in their ability to support patients who have psychosocial difficulties. The issue of protected time and flexibility also needs to be addressed as, while respondents in this study said that lack of time was a barrier to completing the NA, and they also said that the timing of the NA was not always appropriate for patients. In addition, they reported that patients were not always prepared to discuss psychosocial difficulties and attributed this to lack of privacy. If lack of privacy is a barrier, it might be easier for patients and staff to discuss sensitive issues on the telephone as patients treated for breast, colorectal, and endometrial cancer found it easier to discuss sensitive issues over the phone [28–30]. While the clinical practice environments in the UK and Canada differ in their organizational structures and service delivery approaches, these environments have similar barriers with respect to availability of time and need and desire for education and training. Efforts to comprehensively assess cancer patients’ symptoms, problems, and needs will fall short of national guidelines for clinical and psychosocial care in the UK and Canada unless the institutions delivering care are restructured to involve all levels of administrative and clinical staff to adopt a truly patient-centred approach to care. An example of this type of larger scale effort is taking place in Australia, where researchers are developing, implementing, and evaluating a comprehensive program called ADAPT for routine assessment and management of cancer patient distress [31].

### Limitations

Once further recruitment using snowballing in the UK accelerated, it was not possible to know how many nurses had been sent the link to the survey and how many had chosen not to participate. Therefore, we do not know the true response rate for the larger UK population. It is also possible that there was selection bias in the sample, with those who chose to participate and complete the survey having strong opinions about the HNA.

## Conclusion

This study has identified a number of important barriers and facilitators to completion of a NA. That so many busy nurses working in oncology felt so strongly about the implementation of a NA that they were prepared to complete this survey and take the time to elaborate on forced choice responses demonstrates the importance they attach to the topic. They did not dispute the benefit of the NA to patients. However, the challenges they face with introducing the NA as part of everyday practice require investment in training, time, support services, and environment. With the financial pressures facing health services in the UK and Canada, further research is needed to ensure that the NA is conducted at optimum times for those patients who need it to make the best use of patient and staff time.

## References

[1] Canadian Cancer Statistics Advisory Committee (2019) Canadian Cancer Statistics 2019. Canadian Cancer Society, Toronto, ON. Available at: http://www.cancer.ca/en/region-selector-page/?url=%2fen%2fcancer-information%2fcancer-101%2fcanadian-cancer-statistics-publication%2f (accessed January, 2020)

[2] http://www.macmillan.org.uk/documents/aboutus/research/keystats/statisticsfactsheet.pdf (accessed June 2017)

[3] National Cancer Survivorship Initiative (NCSI) (2010) www.dh.gov.uk

[4] Achieving World-Class Cancer Outcomes. A strategy for England 2015-2020. A report of the Independent Cancer Taskforce July 2015

[5] Maddams J, Brewster D, Gavin A, Steward J, Elliot J, Utley M (2009). Cancer prevalence in the United Kingdom: estimates for 2008. Br J Cancer.

[6] Bultz BD, Groff SL, Fitch M, Blais MC, Howes J, Levy K, Mayer C (2011). Implementing screening for distress, the 6th vital sign: a Canadian strategy for changing practice. Psychooncology.

[7] Cancer Action Team (2007) Holistic Common Assessment of Supportive and Palliative

[8] Howell D, Keshavarz H, Esplen MJ, Hack T, Hamel M, Howes J, Jones J, Li M, Manii D, McLeod D, Mayer C, Sellick S, Riahizadeh S, Noroozi H, Ali M, On behalf of the cancer journey advisory group of the Canadian Partnership Against Cancer (2015). A Pan Canadian Practice Guideline: Screening, Assessment and care of psychosocial distress, Depression, and Anxiety in Adults with cancer.

[9] Bruera E, Kuehn N, Miller MJ, Selmser P, Macmillan K (1991). The Edmonton Symptom Assessment System (ESAS): a simple method for the assessment of palliative care patients. J Palliat Care.

[10] Comprehensive Problem and Symptom Screening (COMPASS). Retrieved from https://www.cancercare.mb.ca/export/sites/default/Patient-Family/.galleries/files/your-first-visit-files/COMPASS.pdf

[11] Department of Health (2007) Cancer reform Strategy

[12] NHS England, Cancer Patient Experience Survey (2014) Quality health

[13] Macmillan (2011) Cancer Clinical Nurse Specialist: An evidence review

[14] National Cancer Action Team (2011) www.cquins.nhs.uk/.../NCAT_NCPR_National_Report_2011-12.pdf

[15] Richardson A Finding the time for teams to undertake holistic needs assessment. Poster presentation from Southampton University Hospitals NHS Trust. (Available at http://ncat.nhs.uk/sites/default/files/NCAT%20Poster%20-%20HNA%20and%20lean.pdf)

[16] Tarrant C, Sinfield P, Agarwal S, Baker P (2008). Is seeing a specialist nurse associated with positive experiences of care? The role and value of specialist nurses in prostate cancer care. BMC Health Serv Res.

[17] Carlson LE, Waller A, Mitchell AJ (2012). Screening for distress and unmet needs in patients with cancer: review and recommendations. J Clin Oncol.

[18] Fillion L, Cook S, Blais MC (2011). Implementation of screening for distress with professional cancer navigators. Oncologie.

[19] Bultz BD, Carlson LE (2013). A commentary on “Effects of screening for psychological distress on patient outcomes in cancer: a systematic review.”. J Psychosom Res.

[20] Morse JM, Field PA (1998). Nursing research: the application of qualitative approaches.

[21] Wells M, Cunningham M, Lang H, Swartzman S, Philip J, Taylor L, Thomson J (2015). Distress, concerns and unmet needs in survivors of head and neck cancer: a cross-sectional survey. Eur J Cancer Care.

[22] http://www.macmillan.org.uk/documents/aboutus/research/researchandevaluationreports/throwinglightontheconsequencesofcanceranditstreatment.pdf (accessed June 2017)

[23] Simard S, Thewes B, Humphris G, Dixon M, Hayden C, Mireskandari S, Ozakinci G (2013). Fear of cancer recurrence in adult cancer survivors: a systematic review of quantitative studies. J Cancer Surviv.

[24] Reed SC, Bell JF, Whitney R, Lash R, Kim KK, Bold RJ (2017). Psychosocial outcomes in active treatment through survivorship. Psychooncology..

[25] McLeod D, Esplen MJ, Wong J, Hack TF, Fillion L, Howell D, Fitch M, Dufresne J (2018). Enhancing clinical practice in the management of distress: the Therapeutic Practices for Distress Management (TPDM) project. Psycho-Oncology.

[26] Biddle L, Paramasivan S, Harris S, Campbell R, Brennan J, Hollingworth W (2016). Patients' and clinicians' experiences of holistic needs assessment using a cancer distress thermometer and problem list: a qualitative study. Eur J Oncol Nurs.

[27] Young J, Cund A, Renshaw M, Quigley A, Snowden A (2015). Improving the care of cancer patients: holistic needs assessment. Br J Nurs.

[28] Beaver K, Williamson S, Chalmers K (2010). Telephone follow-up after treatment for breast cancer: views and experiences of patients and specialist breast care nurses. J Clin Nurs.

[29] Williamson S, Chalmers K, Beaver K (2015). Patient experiences of nurse-led telephone follow-up following treatment for colorectal cancer. Eur J Oncol Nurs.

[30] Williamson S, Beaver K, Gardner A, Martin-Hirsch P (2018). Telephone follow-up after treatment for endometrial cancer: a qualitative study of patients’ and clinical nurse specialists’ experiences in the ENDCAT trial. Eur J Oncol Nurs.

[31] Butow P, Shaw J, Shepherd HL (2018). Comparison of implementation strategies to influence adherence to the clinical pathway for screening, assessment and management of anxiety and depression in adult cancer patients (ADAPT CP): study protocol of a cluster randomised controlled trial. BMC Cancer.

